# Cutaneous amoebiasis: a dermatological rarity^☆^

**DOI:** 10.1016/j.abd.2024.04.008

**Published:** 2024-11-26

**Authors:** John Verrinder Veasey, Helena Pladevall Moreira, Mariana de Figueiredo Silva Hafner, Rute Facchini Lellis

**Affiliations:** a Clinic of Dermatology, Santa Casa de São Paulo Hospital, São Paulo, SP, Brazil; b Discipline of Dermatology, Santa Casa de São Paulo School of Medical Sciences, São Paulo, SP, Brazil; c Laboratory of Pathology,Santa Casa de São Paulo Hospital, São Paulo, SP, Brazil

*Dear Editor,*

Cutaneous amebiasis (CA) is a rare infectious disease caused by *Entamoeba histolytica*, a protozoan present worldwide, with higher frequency in patients from the intertropical region.^1^ CA occurs as a primary event when the protozoan is inoculated directly into the skin (surgical procedures, via catheter), and secondary when associated with digestive tract infection through hematogenous dissemination, or by contiguity, mainly in the perianal/vulvar region, in the skin around the drainage of a liver abscess and around colostomies.^1^, ^2^ Secondary events are the most frequent, and cutaneous involvement without intestinal contiguity is rare.^2^

The present case describes a 47-year-old female patient with a history of chronic kidney disease requiring during an acute episode, referred to the Dermatology department with nodular lesions and abscesses, presenting purulent secretion drainage, which appeared primarily at the site of a Shilley catheter implantation in the right subclavian vein four months before. The lesions spread to the abdomen, anterior and posterior thorax, and were painful to palpation. The patient denied systemic symptoms such as fever, unintentional weight loss, and night sweats. Empirical treatment with antimicrobials (ciprofloxacin and cephalexin) was instituted, without improvement of the condition.

On physical examination, several nodules and subcutaneous tumors measuring up to six centimeters in their largest axis were observed, covered by slightly desquamative erythematous-violaceous skin with an ulcerated center, some of which showed spontaneous drainage of purulent secretion (Fig. 1).

**Fig. 1:**
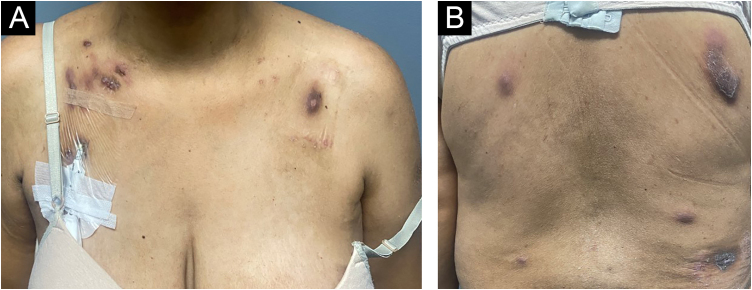
Clinical appearance of patient with cutaneous amebiasis at the first dermatological consultation: nodular lesions and tumors on the abdomen, anterior and posterior thorax covered by slightly desquamative erythematous-violaceous skin; some lesions showed spontaneous drainage of purulent secretion.

Fine-needle aspiration biopsy guided by dermatological ultrasound was performed on a lesion in the posterior thorax (the largest lesion). The collected material was sent to the microbiology laboratory for culture of aerobic, anaerobic and micro bacteria, which did not show growth of bacterial colonies, and to the parasitology laboratory, where direct examination showed structures compatible with cysts and trophozoites of *Entamoeba histolytica* (Fig. 2). A skin biopsy was also performed, and histopathology showed an histiocytic reaction surrounding abscesses in the superficial and deep dermis, with trophozoites of *Entamoeba histolytica* identified on high power microscopy (Fig. 3).

**Fig. 2:**
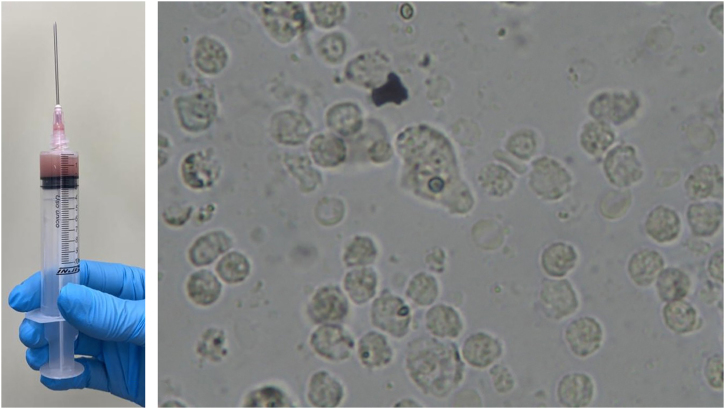
Direct examination of secretion collected by ultrasound-guided puncture (A) showing the parasite *Entamoeba histolytica* in the form of cysts and, in the center of the image (B), trophozoites.

**Fig. 3:**
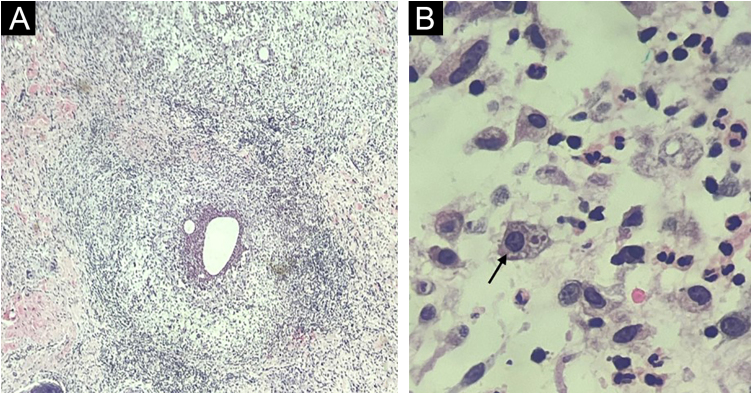
Histopathology. (A) Skin section showing an histiocytic reaction surrounding abscesses in the superficial and deep dermis (Hematoxylin & eosin, ×40). (B) Evidence of *Entamoeba histolytica* trophozoites with similar morphology as on direct examination (Hematoxylin & eosin, ×1000 under immersion oil).

Additional tests were performed to investigate involvement in other organs by the parasites. The parasitological stool examination was negative in three samples for helminths and protozoa, and computed tomography scans of the abdomen and pelvis did not show the presence of a liver or intestinal abscess.

Laboratory tests performed before and after treatment did not show any changes in liver enzymes, either canalicular or transaminases. The patient had normocytic and normochromic anemia related to her underlying disease.

Oral treatment with metronidazole 250 mg every eight hours for 20 days combined with ivermectin 12 mg, in a single dose, was performed. The patient was reevaluated 30 days after the end of metronidazole therapy, showing significant improvement in the lesions, with regression of the nodules and subcutaneous tumors, absence of erythema, and only residual hyperchromia (Fig. 4).

**Fig. 4:**
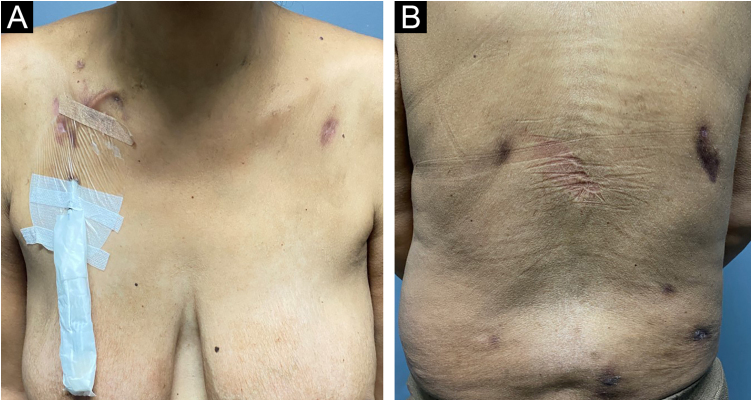
Clinical appearance of the patient with cutaneous amebiasis after treatment with metronidazole and ivermectin, showing only residual lesions.

Amebiasis is a public health problem worldwide, particularly in developing countries. The first reports were probably made by Hippocrates, who discussed dysentery associated with inflammation of the liver.^3^ However, cutaneous involvement is extremely rare. Review studies that assess decades of care and scientific publications show modest numbers of at most 26 cases in children and adults.^3^, ^4^, ^5^

Although the cutaneous lesions can occur anywhere in the body, anogenital ulcers are the main manifestation, and the location has been reported in both children and adults.^3^, ^4^ The trophozoites are continually shed and the surrounding skin undergoes repeated trauma, which is the main mechanism of infection: the amoebae escape from the intestine to the adjacent skin.^2^ Transmission can also occur through contact with contaminated fomites, as well as through sexual intercourse. Hematogenous dissemination occurs when the trophozoites carried by the bloodstream reach the liver – or rarely other organs such as the lungs – and migrate to the skin in a secondary invasion movement.^3^ In the present case, the association with the placement of a Shilley catheter was already evident in the anamnesis, and the absence of evidence of amoebiasis in the complementary exams reinforces this pathogenesis. Subsequent hematogenous dissemination occurred, demonstrated by the presence of disseminated lesions, not only at the site of catheter implantation.

Diagnosis can be easily confirmed both by direct examination of lesion secretion (smear in ulcers, puncture of nodules/abscesses), and by histopathology which can demonstrate *E. histolytica* trophozoites on routine staining (Hematoxylin & eosin).^3^, ^6^ Treatment is carried out with metronidazole in most cases, and other medications such as chloroquine, tinidazole, and emetine may be used, or surgical excision of lesions may be performed.^2^, ^3^ Without a correct diagnosis and immediate treatment, it can result in serious morbidity and even death.^4^ The present patient was treated with metronidazole and ivermectin, and the catheter was also replaced, which did not show any parasites in laboratory tests. The patient showed a good therapeutic response and is being followed at the dermatology outpatient clinic.

## Financial support

None declared.

## Authors' contributions

John Verrinder Veasey: Design and planning of the study; collection of data, or analysis and interpretation of data; drafting and editing of the manuscript or critical review of important intellectual content; collection, analysis, and interpretation of data; intellectual participation in the propaedeutic and/or therapeutic conduct of the studied cases; critical review of the literature; approval of the final version of the manuscript.

Helena Pladevall Moreira: Collection of data, or analysis and interpretation of data; drafting and editing of the manuscript or critical review of important intellectual content; collection, analysis and interpretation of data; intellectual participation in the propaedeutic and/or therapeutic conduct of the studied cases; critical review of the literature; approval of the final version of the manuscript.

Mariana de Figueiredo Silva Hafner: Intellectual participation in the propaedeutic and/or therapeutic conduct of the studied cases.

Rute Facchini Lellis: Collection of data, or analysis and interpretation of data; collection, analysis and interpretation of data; critical review of the literature; approval of the final version of the manuscript.

## Conflicts of interest

None declared.

## References

[1] Eichelmann K., Tomecki K.J., Martínez J.D. (2014). Tropical dermatology: cutaneous larva migrans, gnathostomiasis, cutaneous amebiasis and trombiculiasis. Semin Cutan Med Surg.

[2] Parshad S., Grover P.S., Sharma A., Verma D.K., Sharma A. (2002). Primary cutaneous amoebiasis: case report with review of the literature. Int J Dermatol.

[3] Fernández-Díez J., Magaña M., Magaña M.L. (2012). Cutaneous amebiasis: 50 years of experience. Cutis.

[4] Kenner B.M., Rosen T. (2006). Cutaneous amebiasis in a child and review of the literature. Pediatr Dermatol.

[5] Magaña M.L., Fernández-Díez J., Magaña M. (2008). Cutaneous amebiasis in pediatrics. Arch Dermatol.

[6] Magaña M., Magaña M.L., Alcántara A., Pérez-Martín M.A. (2004). Histopathology of cutaneous amebiasis. Am J Dermatopathol.

